# Incorporation of small extracellular vesicles in sodium alginate hydrogel as a novel therapeutic strategy for myocardial infarction

**DOI:** 10.7150/thno.32637

**Published:** 2019-10-11

**Authors:** Kaiqi Lv, Qingju Li, Ling Zhang, Yingchao Wang, Zhiwei Zhong, Jing Zhao, Xiaoxiao Lin, Jingyi Wang, Keyang Zhu, Changchen Xiao, Changle Ke, Shuhan Zhong, Xianpeng Wu, Jinghai Chen, Hong Yu, Wei Zhu, Xiang Li, Ben Wang, Ruikang Tang, Jian'an Wang, Jinyu Huang, Xinyang Hu

**Affiliations:** 1 Department of Cardiology of The Second Affiliated Hospital, Zhejiang University School of Medicine, Hangzhou, PR China;; 2 Cardiovascular Key Laboratory of Zhejiang Province, Hangzhou, PR China;; 3 Nanjing Medical University, Nanjing, PR China;; 4 Institute of Translational Medicine, Zhejiang University, Hangzhou, PR China;; 5 State Key Laboratory of Silicon Materials, School of Materials Science and Engineering, Zhejiang University, Hangzhou, Zhejiang 310027, PR China;; 6 Center for Biomaterials and Biopathways, Department of ChemistryState Key Laboratory of Silicon Materials, School of Materials Science and Engineering, Zhejiang University, Hangzhou, Zhejiang 310027, PR China;; 7 Department of Cardiology, Affiliated Hangzhou First People's Hospital, Zhejiang University School, Hangzhou, PR China.

**Keywords:** sEVs, alginate, hydrogel, stem cells, myocardial infarction.

## Abstract

Bone marrow mesenchymal stem cell (MSC)-derived small extracellular vesicles (sEVs) have been widely used for treating myocardial infarction (MI). However, low retention and short-lived therapeutic effects are still significant challenges. This study aimed to determine whether incorporation of MSC-derived sEVs in alginate hydrogel increases their retention in the heart thereby improving therapeutic effects.

**Methods:** The optimal sodium alginate hydrogel incorporating sEVs system was determined by its release ability of sEVs and rheology of hydrogel. *Ex vivo* fluorescence imaging was utilized to evaluate the retention of sEVs in the heart. Immunoregulation and effects of sEVs on angiogenesis were analyzed by immunofluorescence staining. Echocardiography and Masson's trichrome staining were used to estimate cardiac function and infarct size.

**Results:** The delivery of sEVs incorporated in alginate hydrogel (sEVs-Gel) enhanced their retention in the heart. Compared with sEVs only treatment (sEVs), sEVs-Gel treatment significantly decreased cardiac cell apoptosis and promoted the polarization of macrophages at day 3 after MI. sEVs-Gel treatment also increased scar thickness and angiogenesis at four weeks post-infarction. Measurement of cardiac function and infarct size were significantly better in the sEVs-Gel group than in the group treated with sEVs only.

**Conclusion:** Delivery of sEVs incorporated in alginate hydrogel provides a novel approach of cell-free therapy and optimizes the therapeutic effect of sEVs for MI.

## Introduction

Cardiovascular disease, including myocardial infarction (MI), is a severe clinical condition. A subsequent cardiac remodeling can further adversely affect heart function after MI even if the prognosis was improved from early reperfusion 1, 2. Over the past decades, stem cell transplantation is in the forefront as a new treatment for MI, and a variety of stem cells are in clinical trials 3-5. Many studies have shown that the therapeutic effect of stem cells is due to the secreted paracrine factors which contain various biologically active substances mediating the communication between cells 6-9. Specifically, the sEVs, a subset of the natural nano-scale lipid bilayer vesicles, contain multiple soluble proteins, mitochondrial DNA, mRNAs, and non-coding RNAs from the parent cells and play an important role in tissue damage repair 10-12. The special role of sEVs might be specifically targeted to recipient cells to trigger changes in downstream signaling pathways and deliver cargos for cell-cell communication. Stem cell-derived sEVs therapy can protect cardiomyocytes against apoptosis, promote angiogenesis, and reduce infarct size. However, due to the high internal pressure of the heart, sEVs therapy still suffers from the problems of short retention and escape into the pulmonary circulation 12-16. Many studies showed that systemically administered sEVs had a short half-life (1-6 h) due to clearance by opsonization and the mononuclear phagocyte system with accumulation in the spleen and liver 17, 18. One study tracked sEVs through gluc signals and reported that transplanted sEVs quickly declined within 72h in hindlimb ischemia treatment 19. Therefore, the efficacy was largely limited by the inevitable rapid clearance of sEVs 20. The approach to improve the retention of sEVs needs to be further investigated.

The ionotropic alginate hydrogel biomaterial has been widely applied as a drug delivery system in tissue engineering and regenerative medicine because of its biocompatibility, non-thrombogenic nature, mild gelation process, and resemblance to the extracellular matrix (ECM) 21. Many studies have shown that sodium alginate hydrogel implant does not cause damage to normal myocardium and increases scar thickness because of its physical support 22. It has been used as a controlled release carrier of multiple bioactive cytokines to enhance self-healing and mediate endogenous regeneration 23, 24. Furthermore, because of encouraging preclinical results, cell-free injectable sodium alginate injection for cardiac injury repair and functional reconstruction is in the clinical research of MI and heart failure. Clinical trials indicated that, in addition to standard medical therapy for the treatment of symptomatic heart failure after MI, sodium alginate hydrogel was effective in providing sustained 1-year benefits in exercise capacity, symptoms, and clinical status for patients 25. However, whether sEVs combined with alginate hydrogel can prolong sEVs retention and enhance therapeutic effects is unknown. In this study, we delivered alginate hydrogel incorporated with MSC-derived sEVs into the infracted myocardium in rats. Compared with sEVs injection alone, sEVs-Gel increased the retention of sEVs in the heart and therefore improved the therapeutic effect after MI.

## Methods

### Animals

All animal experiments were performed in accordance with the Care and Use of Laboratory Animals published by the US National Institutes of Health (NIH Publication No.85-23, revised1996) and were approved by the Institutional Animal Care and Use Committee of Zhejiang University. Male Sprague-Dawley (SD) rats (approx, 200 g) were acquired from Zhejiang Chinese Medical University. Animals were fed in a standard laboratory with controlled room temperature (22 ± 1°C), humidity (65-70%), and a 12:12-h light-dark cycle and free access to food and water.

### Preparation of MSCs

Bone marrow was extracted from 4-week old SD rats, which were sacrificed by intraperitoneal injection of pentobarbital sodium (Merck). Tibias were cut off and rinsed three times with Dulbecco's modified eagle medium (DMEM, Thermo) and the rinsing solution was centrifuged to obtain bone marrow. After removing the supernatant, cells were resuspended and cultured in MSCs culture medium (90% (v/v) low glucose DMEM, 10% (v/v) FBS, 1% (v/v) Penicillin-Streptomycin solution) at 37 °C and 5% CO_2_ in a humidified atmosphere until confluent. The adherent cells were digested with 0.25% trypsin containing 0.02% EDTA (Genom, China) and cultured for 3-5 passages. MSCs were identified by flow cytometry as described previously 26. MSCs were propagated for 3-5 passages. Subsequently, conditioned media were collected to isolate sEVs.

### Isolation, Characterization of sEVs

sEVs were isolated as previously reported 27, 28. MSCs were cultured to 80% confluence in the complete medium washed three times with PBS, and subsequently cultured with low glucose DMEM and 1% (v/v) Penicillin-Streptomycin solution for 48 h. The conditioned medium was collected and centrifuged at 500 g for 10 min to remove cells followed by 10000 g for 30 min to remove apoptotic bodies and cell debris. The supernatant was centrifuged at 120000 g for 70 min. The supernatant was discarded, and the pellet was resuspended in PBS and centrifuged 120000 g for another 70 min to purify sEVs, which were quantified by using the Bradford Protein Assay Kit (Beyotime, Shanghai, China). Usually 50 μg of sEVs were released from about two million MSCs in 48 h. Western blotting was used to identify surface makers of sEVs including CD63 (ab193349, Abcam, Cambridge, MA, USA), GM130 (ab52649, Abcam, Cambridge, MA, USA), and Alix (sc53540, Santa Cruz Biotechnology, Shanghai, China). Transmission electron microscopy (TEM) was used to determine particle morphology and the particle size distributions of isolated sEVs suspended in PBS was determined using ZetaView (Particle Metrix, Germany).

### Preparation of alginate hydrogel incorporating sEVs

Sodium alginate powder (Aladdin, Shanghai, China) was dissolved in PBS to form 2% (wt/vol) sodium alginate solution at room temperature. The sEVs collected as described above were added into the alginate solution to stir and mix evenly. Calcium chloride solution (0.5%、1%、2%, wt/vol) was added to alginate solution at a volume ratio of 1:4 and incubated at 37 °C for 10 min to form alginate hydrogel incorporating sEVs. An AR-G2 rheometer was used to test their rheological behavior.

### sEVs release from alginate hydrogel

For release studies, 100 μL of alginate hydrogel incorporating 80 μg of sEVs were incubated in 200 μL PBS (37 °C, 5% CO_2_ atmosphere). The supernatant was removed and fresh PBS was added every two days. The number of sEVs released into the medium was determined by the Bradford Protein Assay Kit.

### Scanning electron microscopy (SEM) and Transmission electron microscopy (TEM)

The prepared sodium alginate hydrogel incorporating sEVs-Gel were fixed with 10% formalin solution at 37 °C overnight. Subsequently, samples were washed with PBS three times and were sequentially dewatered by 10%, 30%, 50%, 70%, and 100% ethanol solution. Finally, the samples were subjected to critical point drying and were observed by KYKY EM-3200 SEM. sEVs were also stained with uranyl acetate for 30 min at room temperature and loaded onto 200-mesh copper electron microscopy grids for imaging using a H7500 transmission electron microscopy (Hitachi, Japan).

### MI model and treatment

MI was surgically induced as described previously 29. Male SD rats were abdominally anesthetized with pentobarbital sodium (50 mg/kg) and ventilated with a rodent ventilator. The chest was opened and the heart was exposed. An 8-0 nylon suture was used to ligate left anterior descending coronary artery permanently, and the infarction was confirmed by observing the blanching of the myocardium in the affected area. Left anterior descending coronary artery was not ligated in the sham group rats. Thirty minutes after ligation, animals in the sEVs, Gel, and sEVs-Gel groups were intramyocardially injected with a suspension containing 80 μg of sEVs in 100 μL PBS, 100 μL of alginate hydrogel, and 100 μL of alginate hydrogel incorporating 80 μg of sEVs, respectively. The MI group received the same volume of PBS. The suspensions were injected at four sites in the border of the infarct area and one site in the infarct area.

### sEVs labeling and fluorescence imaging

sEVs suspended in PBS were labeled with 1:400 diluted DiR (Thermo Fisher Scientific) for 30 min at 37°C and dye was removed to purify sEVs using exosome spin columns (MW 3000; Thermo Fisher Scientific). The rats were sacrificed, and the heart, liver, spleen, lungs, and kidneys were acquired to examine the distribution of sEVs in various major organs. Near-IR fluorescence images were acquired using the IVIS Spectrum System (PerkinElmer), and fluorescence intensity of main organs between the sEVs and sEVs-Gel groups was compared.

### Assessment of cardiac function

Echocardiographic studies were performed using the Vevo 2100 Imaging System (VisualSonics, Inc). Echocardiograms were captured under isoflurane anesthesia before surgery, within 4 h, and at day 28 post-surgery. Left ventricular ejection fraction (LVEF), left ventricular fraction shortening (LVFS), left ventricular end diastolic diameter (LVIDD), and left ventricular end systolic diameter (LVIDS) were analyzed using the Vevo 2100 workstation software.

### Histology

Twenty-eight days post-surgery, rats were euthanized and hearts were quickly excised. Hearts were dehydrated in 30% sucrose solution and embedded in Tissue-Tek OCT compound to snap freeze in liquid nitrogen. The samples were cut into 7 μm slices from apex to mid-papillary (800 μm intervals) and stained with Masson's trichrome (Solaribio, Beijing, China) to examine the infarct size and wall thickness of the infarct zone. The infarct area was calculated by the sum of the endocardial and epicardial length of the infarct zone divided by the total length of the endocardial and epicardial left ventricle, and the wall thickness of infarct zone was averaged from three to five measurements of scar thickness using Imaging Pro Plus software.

### Immunofluorescence Staining

Primary antibodies, anti-CD31 (ab24590, Cambridge, MA, USA) and α-smooth muscle actin (ab7817, Cambridge, MA, USA), were used to observe angiogenesis. Mouse anti-CD68 (ab955, Abcam, Cambridge, MA, USA) and rabbit anti-CD206 (ab64693, Abcam, Cambridge, MA, USA) were used to detect inflammation. Wheat germ agglutinin (WGA, Thermofisher Scientific, USA) and rabbit anti-TnI (ab47003, Abcam, Cambridge, MA, USA) were used to examine viable myocardium in the infarct area. The sections were then incubated with corresponding fluorescentdye-conjugated secondary antibodies (ab96875, Abcam; ab96876, Abcam; ab96892, Abcam; ab150073, Abcam) and nuclei were counterstained with DAPI (Vector Laboratories, Burlingame, CA, USA) according to manufacturer's instruction. We also used TUNEL kit (Roche Applied Science, Indianapolis, IN, USA) to evaluate cell apoptosis. The TUNEL-positive cardiac cell percentage was calculated by the ratio of the TUNEL-positive nuclei to the total nuclei in at least five randomly selected fields in each heart and four hearts were evaluated per group. The proportion of M2 macrophages was the ratio of CD206 positive cells to CD68 positive cells in at least five randomly selected fields in each heart and three hearts were evaluated per group. CD31- and α-SMA-positive vessels were counted in each field divided into the border, infarct, and remote zones and analyzed in three to five randomly selected fields in each sample; three samples were evaluated per heart, and five hearts were evaluated per group. Troponin-positive cardiomyocytes proportion in the infarct area was calculated by the ratio of the troponin-positive area to the total infarct area in at least five randomly selected fields in each heart and five hearts were evaluated per group.

### RNA extraction and quantitative real-time PCR

RNA was extracted from sEVs by TRIzol (Invitrogen Life Technologies) and a NanoDrop spectrophotometer (NanoDrop Technologies, ThermoFisher Scientific) was used to evaluate nucleic acid quality and quantity. Then miRNAs were reverse transcribed by miRNAs reverse transcription kit (RiboBio, China) using SYBR Premix Ex Taq (TAKARA BIOTECHNOLOGY CO., Dalian, China). The miRNA expression levels were normalized to U6 expression.

### Tube formation of HUVECs (human umbilical vein endothelial cells)

A Matrigel tube-formation assay was performed to assess angiogenesis *in vitro* 30. Briefly, cells (1×10^5^) were seeded in the lower chamber and 100 μL of hydrogel or sEVs incorporated in hydrogel was placed in the upper chamber in a Transwell (Corning, 8 μm) co-culture system. The cells were cultured in low glucose DMEM medium with 1% (v/v) Penicillin-Streptomycin solution at 37 °C and 5% CO_2_ in a humidified atmosphere. Fresh medium was added after day 1 and cells were harvested after incubation for 48 h. HUVECs were plated at 1×10^4^ cells/well in 96-well plates pre-coated with growth factor-reduced Matrigel (BD, San Jose, CA, USA). After 5 h, formed tubes were observed and the tube length was quantified.

### Western blotting

sEVs extracted above were added to 5×SDS gel loading buffer [30 mM Tris-HCl (pH 6.8), 2% SDS, 0.05% bromphenol blue, 12.5% glycerol, and 2.5% mercaptoethanol] and then boiled for 30 min. Then 20 μg of sEVs were added into each lane on 10% SDS polyacrylamide gels and the mixtures were resolved for Western blotting analysis. The samples were blotted onto PVDF membranes (Millipore, Boston, MA, USA), which were blocked with 5% milk for 1 hour at room temperature and then probed with relevant primary antibodies of CD63 (ab193349, Abcam, Cambridge, MA, USA), GM130 (ab52649, Abcam, Cambridge, MA, USA), and Alix (sc53540, Santa Cruz Biotechnology, Shanghai, China) at 4°C overnight. Subsequently, membranes were reacted with horseradish peroxidase-conjugated secondary antibodies for 1 h at room temperature and observed using the ECL kit chemiluminescence reagents (Millipore, Billerica, MA, USA). Similarly, tissue proteins were isolated from hearts with RIPA lysis buffer and 20 μg of proteins per sample were analyzed using primary antibodies against hepatocyte growth factor (HGF, ab83760, Abcam, Cambridge, MA, USA), vascular endothelial growth factor (VEGF, ab64154, Abcam, Cambridge, MA, USA), and platelet derived growth factor BB (PDGF-BB, ab16829, Abcam, Cambridge, MA, USA). Other procedures were the same as described above.

### Statistical Analysis

Results were expressed as mean ± SD. Significant differences between two groups were determined by the Student's t-test, and One-way ANOVA test was used for multiple comparisons. P < 0.05 was considered statistically significant. Statistical calculations were carried out using GraphPad Prism 6.0.

## Results

### Characterization of MSC-derived sEVs

sEVs were obtained from the culture medium of MSCs by multiple centrifugations. The presence of lipid bilayer sEVs was demonstrated using TEM; its particle size was analyzed with ZetaView and determined to be about 100 nm (Figure S1A-B). Western blotting (Figure S1C) also detected the expression of sEVs positive markers CD63 and Alix, and negativ maker GM130, further confirming the successful extraction of sEVs.

### Preparation of sEVs incorporated in sodium alginate hydrogel (sEVs-Gel)

**Figure 1:**
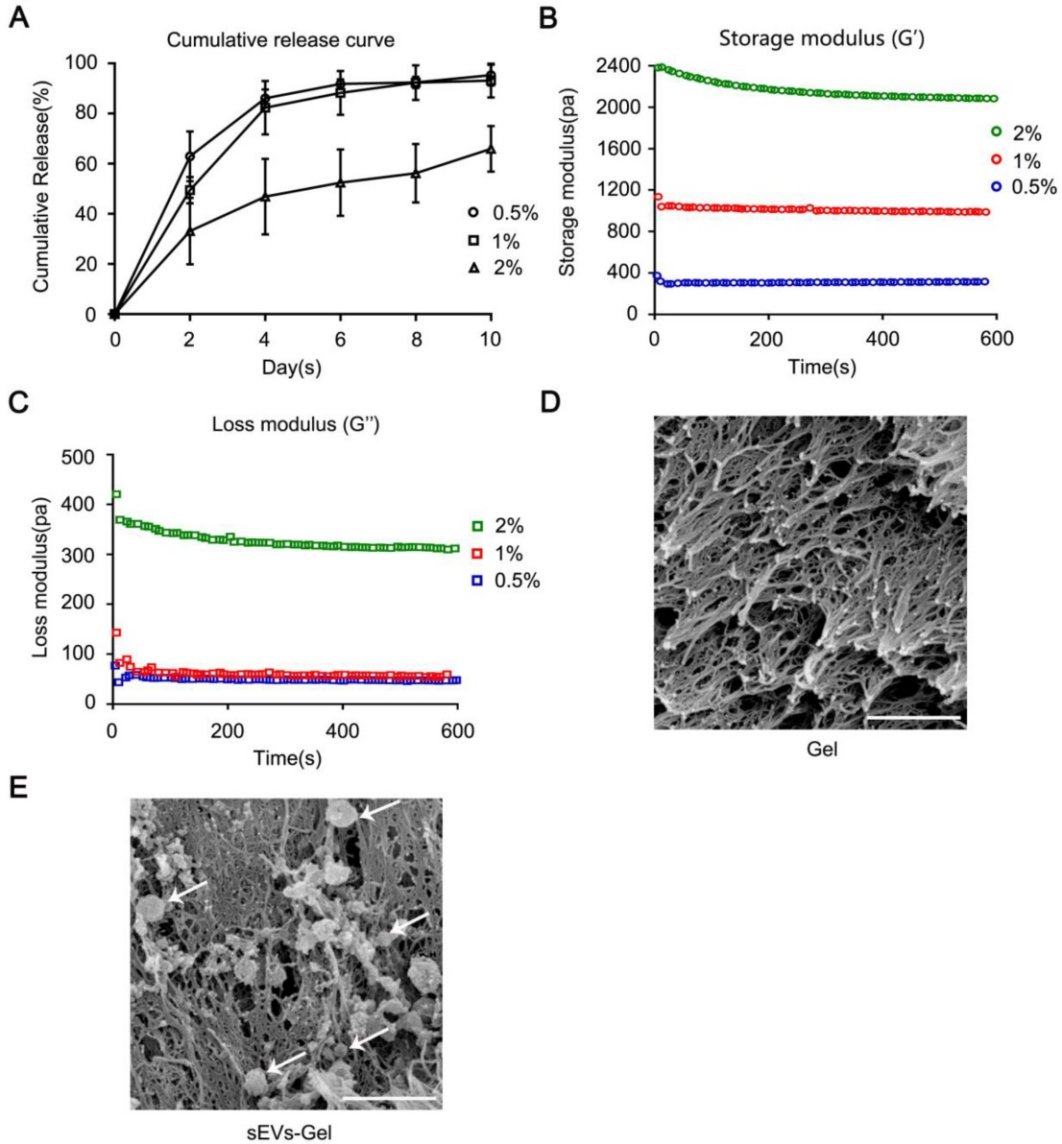
Rheological behavior and sustained release analysis of MSC-derived sEVs incorporated in alginate hydrogel. (A) The cumulative release profile of sEVs-containing hydrogel *in vitro* was analyzed by the Bradford Protein Assay Kit. (B, C) Rheological behavior of hydrogel incorporating sEVs was evaluated via AR-G2 rheometer. (D, E) Scan electron micrographs of hydrogel represent its porous structure of scaffold and the morphology of sEVs loaded in the hydrogel (bar = 500 nm).

To determine the optimal sodium alginate hydrogel system, we examined the release ability of sEVs and rheology of hydrogel using different concentrations of calcium chloride solution (0.5%, 1%, 2% wt/vol). First, the sEVs were blended with 2% (wt/vol) alginate sodium solution followed by incubation with different concentrations of calcium chloride solution at 37 °C for 10 min to prepare the hydrogel incorporating sEVs. To detect and quantify the sEVs controlled release ability, 100 μL of hydrogel with 80 μg of sEVs was immersed in 200 μL PBS and was refreshed with the same amount of PBS every two days. The sEVs released from the hydrogel into PBS were collected every two days and quantified using the Bradford Protein Assay Kit. As shown in Figure 1A, the hydrogels with 0.5% and 1% calcium chloride solutions released the sEVs in about 10 days with a strong burst releasing effect evident in the first few days. On the other hand, the hydrogel with 2% calcium chloride solution released only a part of the total sEVs in 10 days.

In MI, sudden blockage of coronary blood flow leads to cardiomyocyte damage and resultant necrosis accompanied by inflammatory infiltration mainly occurring in the first week. sEVs therapy plays an important role in reducing cardiomyocyte death, resulting in preserved cardiac function and anti-inflammatory effect 10. Based on our results, we reasoned that the hydrogel with 0.5% and 1% calcium chloride solutions might be more effective for treating MI compared with the hydrogel prepared with 2% calcium chloride solution 2.

Next, the rheological property of sodium alginate hydrogel was analyzed. Figure 1B-C show that the storage modulus (G') and the loss modulus (G''). Also, hydrogel composed of 1% CaCl_2_ and 2% alginate sodium solution exhibited a G' value between 400-1800 Pa which is considered a proper G' value in cardiac tissue engineering 31. The hydrogel with 0.5% CaCl_2_ was too soft (G'≈300 Pa) and with 2% CaCl_2_ was too hard (G'≈2000 Pa) to be suitable for transplantation. Based on these two characteristics, we chose 1% CaCl_2_ and 2% alginate sodium solution for the synthesis of hydrogel because of its appropriate G' value and the release curve suitable for treatment. The interconnected porous structure of scaffold and the morphology of sEVs loaded in the gel were examined by scanning electron microscopy (Figure 1D-E).

### sEVs-Gel boosts the retention of sEVs in the heart

**Figure 2:**
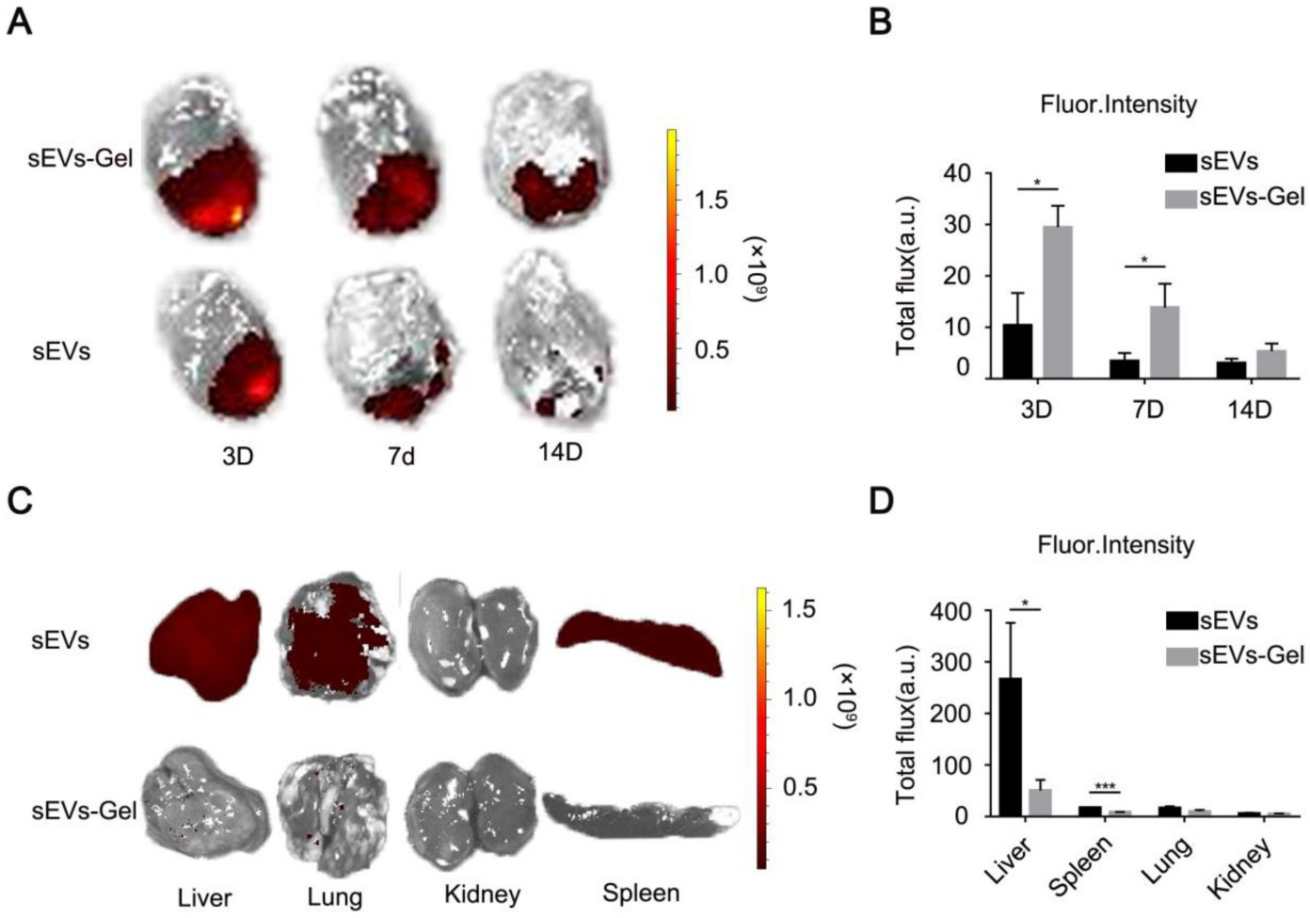
Incorporation of sEVs in hydrogel promote their retention in the heart. (A) Representative ex vivo fluorescence imaging of MI rat hearts at day 3, 7, and 14 after transplantation of hydrogel incorporating sEVs or sEVs alone. (B) Quantitative analysis of fluorescence intensities of rat hearts after transplantation of hydrogel incorporating sEVs or sEVs alone. n=3 for each group. *P < 0.05. (C) Representative ex vivo fluorescence imaging of dissected organs at day 3 after treatments. (D) Quantitative analysis of fluorescence intensities of dissected organs at day 3 after transplantation of hydrogel incorporating sEVs or sEVs alone. n=3 for each group. *P < 0.05;***P <0.001.

The effect of sEVs for treating myocardial infarction was curtailed by only a small number of sEVs remaining in the infarct area 12. Hydrogel, due to its viscosity and hardness, provides a natural matrix barrier to lock sEVs and prevents its rapid loss. Here, we incorporated sEVs in alginate hydrogel, which, due to its hydrophilic and porous features, serves as a temporary repository for the continuous release of sEVs into the infarct heart. To assess whether alginate hydrogel helped to retain sEVs in the heart and hence significantly improved their utilization we labeled sEVs with lipophilic carbocyanine DiR for tracking. The labeled sEVs were intramyocardially injected with or without alginate hydrogel, and their retention was then analyzed by *ex vivo* imaging using the IVIS system. The results at day 3 and 7 post injection revealed a stronger fluorescent signal (representing DiR-labeled sEVs) in the sEVs-Gel-treated hearts compared with those treated with sEVs only (Figure 2A-B), suggesting that hydrogel enhanced sEVs retention in the injured heart. At day 14, there was a trend of high fluorescence intensity in the sEVs-Gel group, but no significant difference was found between the two groups. Furthermore, we assessed fluorescent signals in the liver, spleen, lungs and kidneys at day 3. There was a significantly less fluorescent signal in the liver and spleen in the sEVs-Gel group compared with the sEVs group (Figure 2C-D), indicating that hydrogel indeed retained sEVs in the heart.

### sEVs-Gel protects cardiac cells against apoptosis

**Figure 3:**
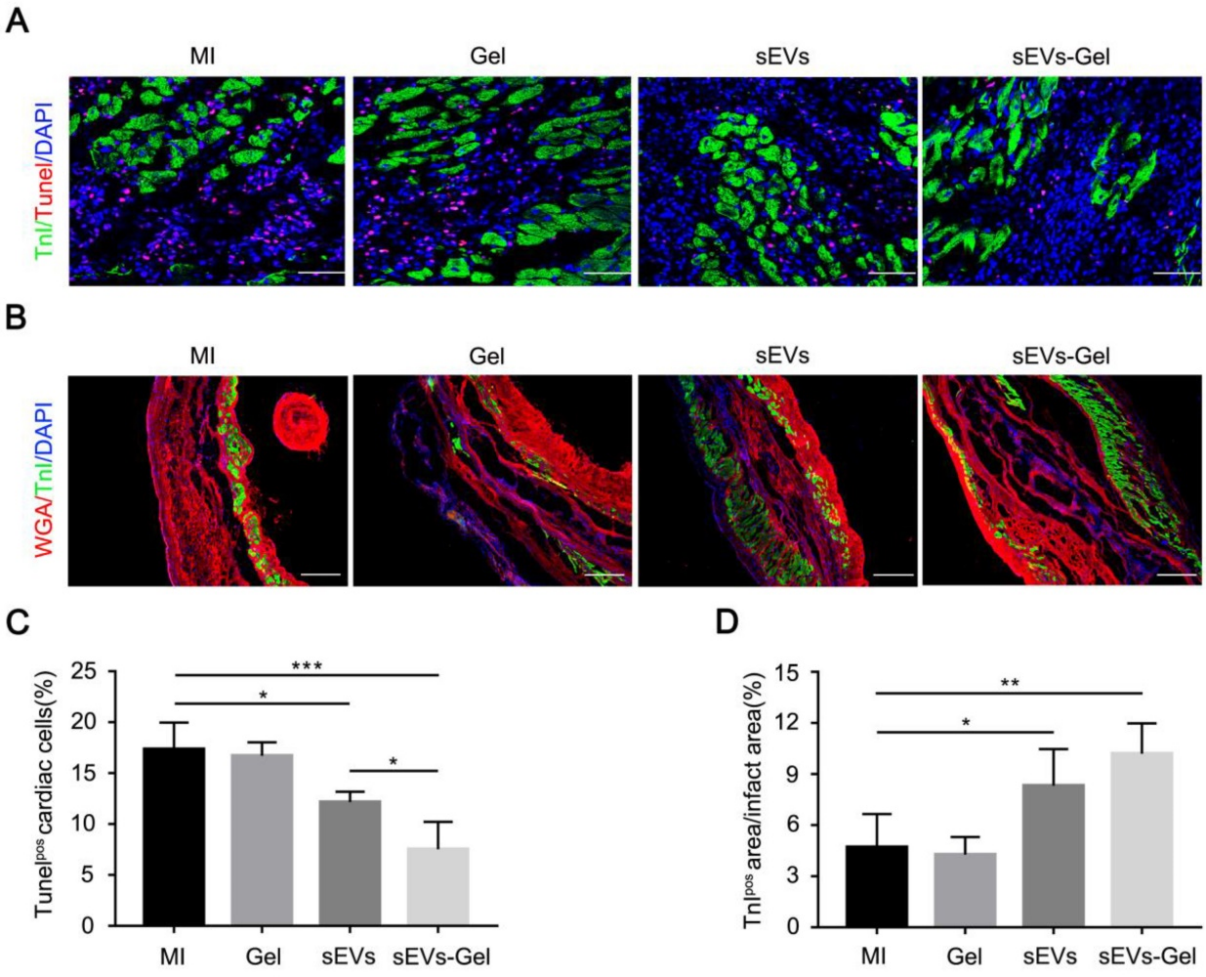
sEVs-Gel reduces apoptosis in the border area and increases the survival of cardiomyocytes in the infarct area. (A) Representative apoptotic cells at day 3 by TUNEL staining in the border area. Bar=50 μm. (B)The infarct area was stained with wheat-germ agglutinin (red), troponin (green) and DAPI (blue). Bar=200 μm. (C) Quantitative analysis of TUNEL-positive cells in the border zone. n=4 for each group. *P < 0.05; ***P < 0.001. (D) Quantitative analysis of TnI-positive area proportion. n=5 for each group. *P < 0.05; **P < 0.01.

sEVs play an important role in the anti-apoptotic process. We, therefore, compared the expression of miRNAs related to anti-apoptosis and pro-angiogenesis in sEVs derived from MSC cells and H9C2 cells. We found that the expression levels of miRNA 19a-3p, 126a-3p, 29-3p, 21-5p, 210-3p, 132-3p were higher in sEVs derived from MSCs (Figure S2). To assess whether enhanced sEVs retention exerted more beneficial effects, we analyzed possible cardiac anti-apoptosis. TUNEL staining performed at day 3 indicated that the percentage of apoptotic nuclei in the border zone was markedly decreased in sEVs and sEVs-Gel groups compared with the MI group, while the sEVs-Gel group exhibited more enhanced anti-apoptotic effect than the sEVs group (Figure 3A and C). Also, there were more viable cardiomyocytes in sEVs and sEVs-Gel groups as compared with MI and Gel groups at day 28 (Figure 3B and D). However, there were no significant differences in anti-apoptosis and the survival of cardiomyocytes between the Gel group and the MI group.

### sEVs-Gel alleviates inflammation through polarizing M2 macrophages as effectors of cardioprotection

**Figure 4:**
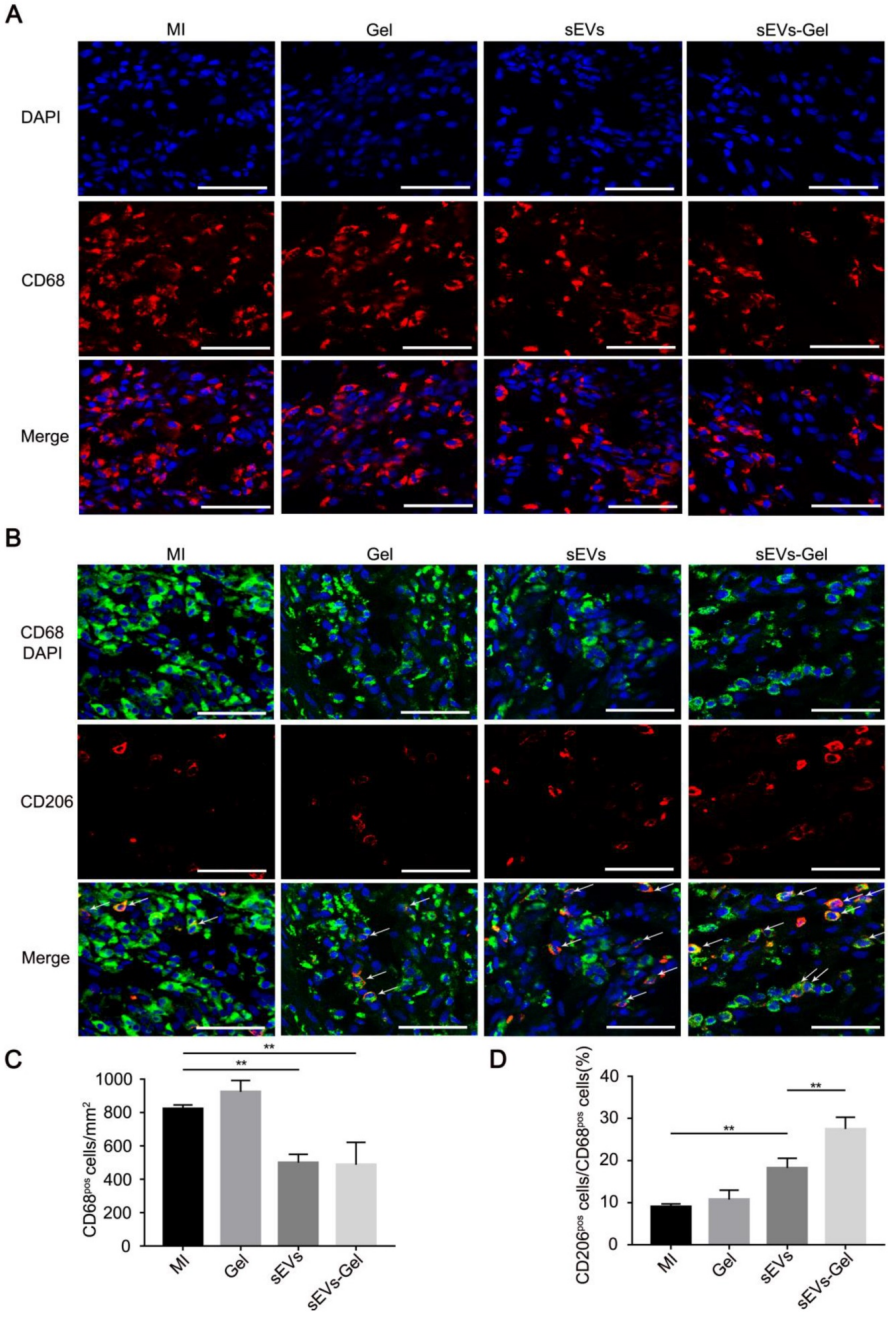
Evaluation of inflammation in the infarct area. (A) Immunofluorescence staining of CD68-positive macrophages at day 3. Bar = 50 μm. (B) Immunofluorescenc staining of CD68- and CD206-positive macrophages at day 3. Bar = 50 μm. (C) Quantitative analysis of CD68-positive cells/mm^2^. n=3 for each group. **P < 0.01. (D) Quantitative analysis of the ratio of CD206 to CD68. n=3 for each group. **P < 0.01.

Although the inflammatory cascade plays an important role in response to cardiac injury, excessive inflammatory reactions are not conducive to heart repair. M1 macrophages are a proinflammatory subtype overexpressing inflammatory genes whereas M2 polarized macrophages are beneficial mediators. Whether the sEVs exerted an anti-inflammatory effect that was improved by sodium alginate hydrogel was not known 32, 33. Our study showed that the number of CD68+ macrophages was decreased in the sEVs-treated group compared with the MI or Gel-treated groups and the proportion of M2 macrophages was increased compared to the MI group (Figure 4A-D). Furthermore, sEVs-Gel treatment decreased the number of CD68+ macrophages similar to the sEVs group but increased the proportion of M2 macrophages compared to the sEVs group, suggesting that sEVs-Gel treatment enhanced macrophages from M1 to M2 phenotype at day 3. Besides, we examined the proportion of M2 macrophages at day 7 and found no significant difference among four groups (Figure S3).

### sEVs-Gel promotes angiogenesis

**Figure 5:**
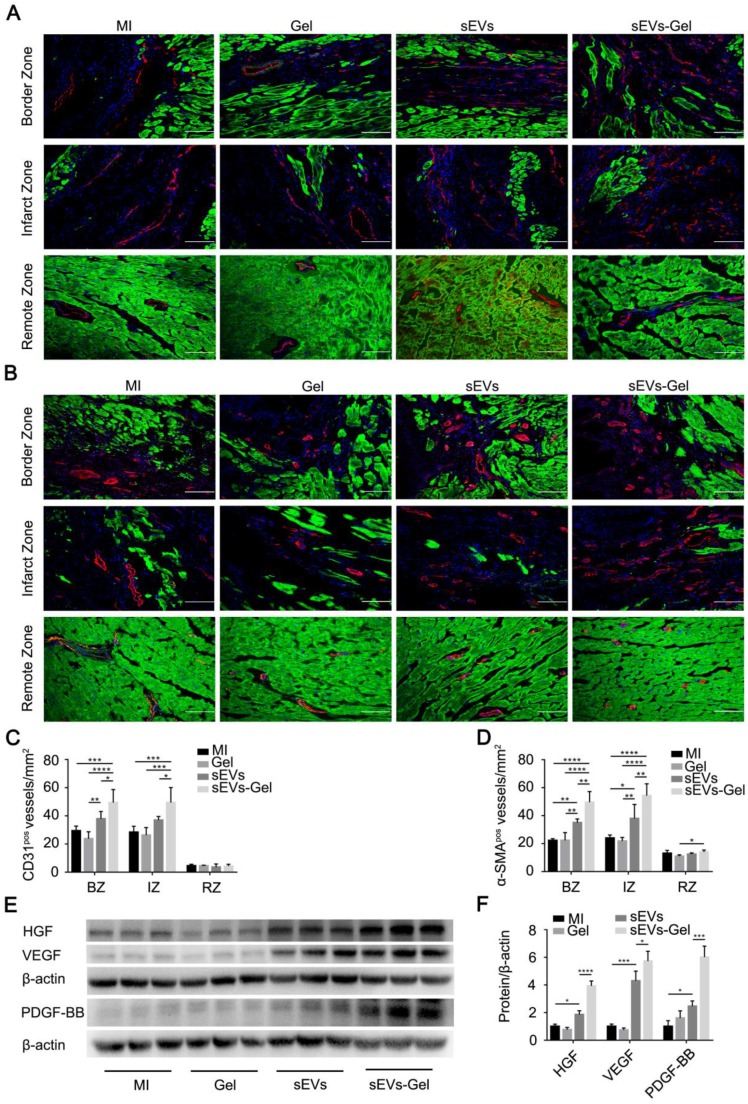
Evaluation of angiogenesis after MI. (A) Immunofluorescence staining of CD31 in the border zone, infarct zone and remote zone at 4 weeks after MI. CD31 (red); troponin (green); DAPI (blue). Bar=100 μm. (B) Immunofluorescence staining of α-SMA in the border, infarct, and remote zones at 4 weeks after MI. α-SMA (red); troponin (green); DAPI (blue). Bar=100 μm. (C) Quantitative analysis of CD31-positive area density. n=5 for each group. *P < 0.05; **P < 0.01; ***P < 0.001; ****P < 0.0001. (D) Quantitative analysis of α-SMA-positive area density. n=5 for each group. *P < 0.05; **P < 0.01; ****P < 0.0001. (E) Expression of the proteins HGF, VEGF, and PDGF-BB was evaluated by Western blotting. β-actin levels served as a control. (F) Protein levels were quantified by densitometry analysis. n=3 per group. *P < 0.05; ***P < 0.001; ****P < 0.0001.

Following MI, the blood supply in the heart is continuously blocked resulting in the irreversible damage to cardiac cells due to ischemia and hypoxia. Regeneration of blood vessels is needed to rescue the ischemic cascade and is associated closely with ventricular remodeling. Therefore, we evaluated the therapeutic pro-angiogenesis process in the border, infarct, and remote zones at day 28 after MI. The density of the vessels with CD31-positive staining in the sEVs-Gel group was higher than the other three groups in the border and infarct zones. There was no significant difference between the MI and Gel groups in the border and infarct zones, and the density of the vessels positive for CD31 was higher in the border zone of the sEVs group than the Gel group (Figure 5A-C). Also, α-SMA staining-positive vascular density in the sEVs-Gel group was significantly higher than the other three groups in the border and infarct zones, while the sEVs group had higher number of α-SMA-positive vessels than MI and Gel groups in the border and infarct zones (Figure 5B-D). Concomitantly, there was no significant difference in the remote zone among all four groups in the number of CD31-positive vessels. α-SMA staining positive vascular density in the sEVs-Gel group was higher than the Gel group in the remote zone. Furthermore, we also assessed the pro-angiogenesis cytokines in infarcted hearts by Western blotting at day 28 after MI. The protein levels of HGF, VEGF, and PDGF-BB were higher in the sEVs group than the MI group, while they were significantly higher in the sEVs-Gel group than in the sEVs group (Figure 5E-F). To evaluate the effect of sEVs-Gel on tube formation *in vitro*, HUVECs pretreated with sEVs or sEVs-Gel were seeded on Matrigel. sEVs remarkably enhanced tube formation compared to the DMEM or hydrogel-treated groups which was considerably increased with the treatment of sEVs incorporated in hydrogel (Figure S4A-B).

### sEVs-Gel promotes the reparative potency of sEVs

**Figure 6:**
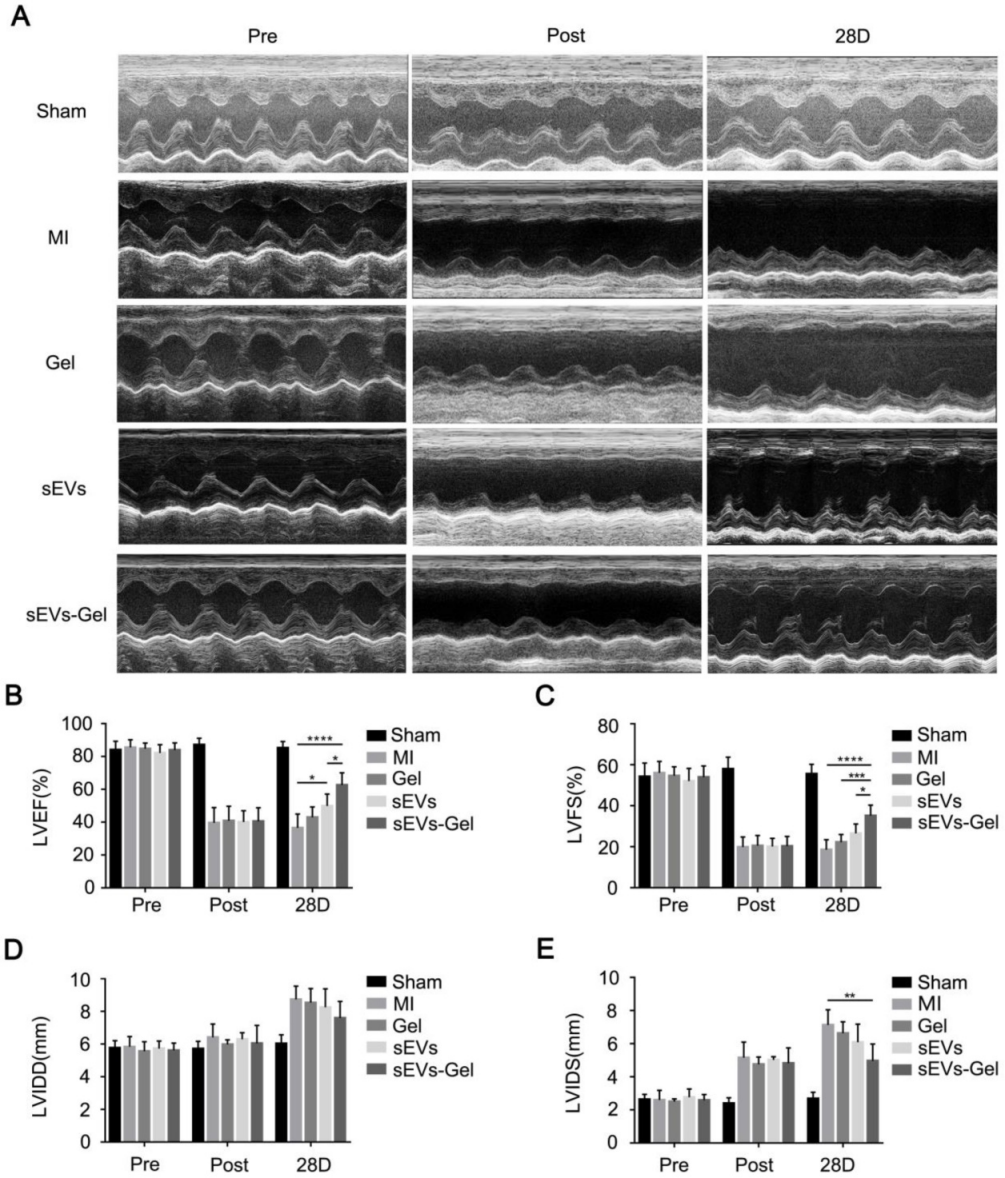
sEVs-Gel treatment enhances cardiac function in rats with MI. (A) Representative echocardiography images of sham group, MI group, Gel group, sEVs group, and sEVs-Gel groups. (B, C) Left ventricular ejection fraction and left ventricular fraction shortening were measured by echocardiography. n=6 animals per group. *P < 0.05; ***P < 0.001; ****P < 0.0001. (D, E) Analysis of left ventricular end diastolic diameter and left ventricular end systolic diameter. n=6 animals per group. **P < 0.01.

To assess whether the enhanced biological effects (anti-apoptosis, pro-angiogenesis, and anti-inflammatory effect) augmented by sodium alginate hydrogel could result in favorable cardiac function, echocardiography was performed before, immediately, and 28 days after MI. The sEVs themselves could ameliorate LVEF at day 28 after injection compared with the MI group (Figure 6A-B). Furthermore, sEVs-Gel delivery significantly improved cardiac function as indicated by a higher LVEF and LVFS as compared with either sEVs or Gel treatment (Figure 6A-C). Although there was no significant difference in LVIDD of each group at day 28 after MI, the sEVs-Gel group had shorter LVIDS than the MI group (Figure 6D-E).

**Figure 7:**
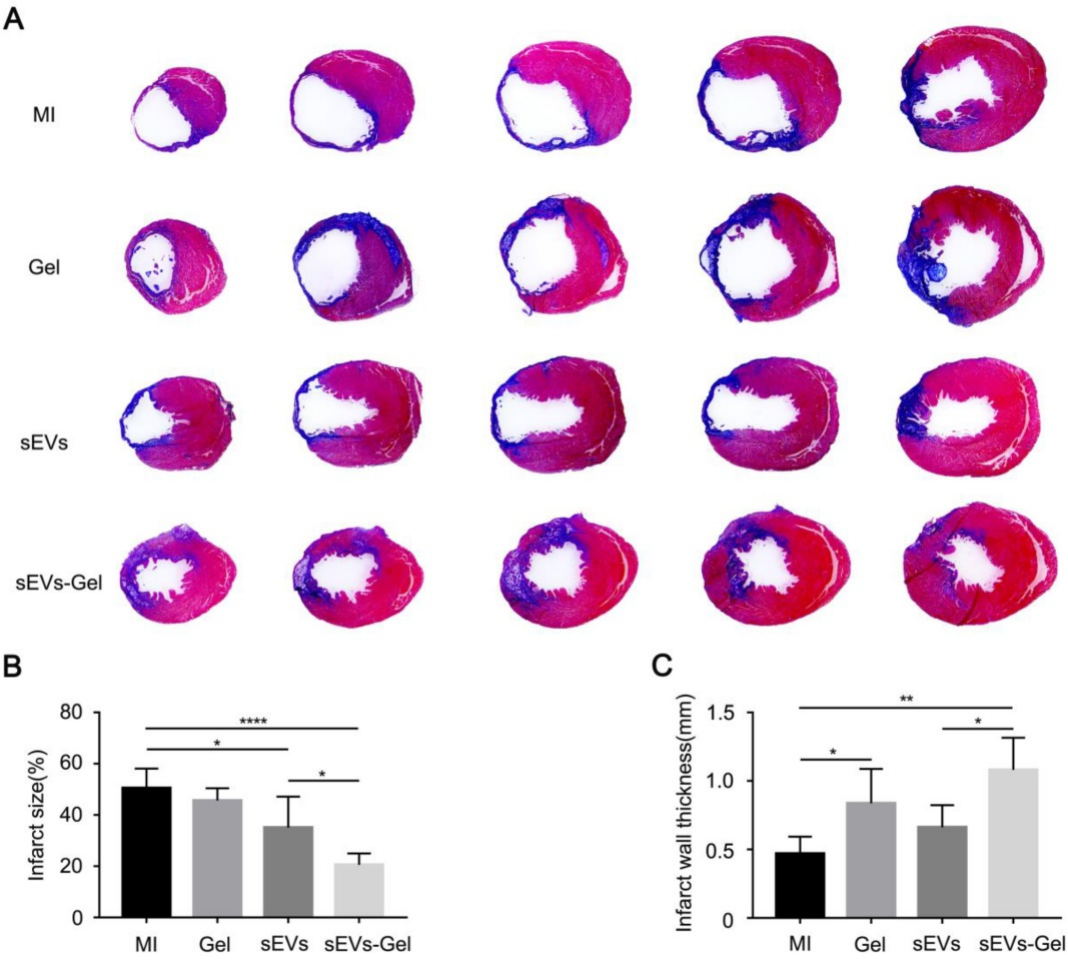
sEVs-Gel delivery reduces infarct size. (A) Representative Masson's trichrome staining images in MI group, Gel group, sEVs group, and sEVs-Gel groups (blue, scar tissue; red, viable myocardium). (B) Quantitative analysis of infarct size. n≥5 animals per group. *P < 0.05; ****P < 0.0001. (C) Quantitative analysis of infarct wall thickness. n≥5 animals per group. *P < 0.05; **P < 0.01.

To further confirm the cardiac function improved by sEVs-Gel, its effects on the heart were examined histologically at day 28 after MI. When heart sections were examined by Hematoxylin-Eosin staining, no hydrogel was visible in the hearts of MI and sEVs group, while residual hydrogel was found in Gel and sEVs-Gel groups (Figure S5). As a polysaccharide complex, hydrogel itself was filled with necrotic tissue, resulting in increased thickness of the infarct area. Masson's trichrome staining was used to visualize the infarct size (Figure 7A). The percentage of fibrosis in the left ventricle, indicating the total infarct size, was notably increased and the scar thickness was significantly decreased in the MI group. The sEVs group exhibited a significantly decreased infarct size compared with the MI group, while sEVs-Gel further reduced the infarct size compared to the sEVs group (Figure 7B). The wall thickness of the infarct area in the sEVs-Gel group was thicker than that in MI and sEVs groups (Figure 7C).

## Discussion

In this study, we provided evidence that the retention of sEVs in the heart was improved by alginate hydrogel, sEVs-Gel, which prevented fast diffusion of sEVs out of the heart. The hydrogel, as a polymer chain network, temporarily stored sEVs and allowed its slow release better mediating myocardial damage repair. Thus, compared to the application of sEVs or hydrogel alone, post-MI intramyocardial injection the hydrogel incorporated with sEVs (sEVs-Gel) enhanced the reparative potency of sEVs in pro-angiogenesis, reducing fibrosis and improving cardiac function after MI. The alginate hydrogel had properties similar to the cardiac matrix and supported the infarcted LV wall tissue by increasing the scar thickness to reduce LV wall stress. Our results provide a novel approach of cell-free therapy in which the repair of cardiac function after MI is reinforced from the sustained release of therapeutic sEVs.

sEVs are considered a key component of the paracrine factors extended by stem cells. Similar to stem cells, sEVs, by mediating cell-cell micro-communication, can protect the myocardial tissue from cell death significantly improving the cardiac function. Comparable with stem cell therapy, sEVs have similar beneficial effects in cardiomyocyte protection, angiogenesis, immune regulation, and tissue repair 34.

Comparing with stem cells, sEVs have similar beneficial activity accompanied by low immunogenicity and low potential ectopic engraftment or teratoma formation risk 35, 36. An additional advantage of sEVs is, these nanoscale extracellular vesicles can be chemically or biologically modified to enhance and broaden their therapeutic effects 37. Therefore, cell-free therapy of sEVs has become a promising approach for myocardial repair.

However, the lower retention of sEVs in the heart presents a major challenge. Even if the concentration of sEVs is increased in the local injection, there are barely any residual sEVs in the heart due to the high blood flow circulation. Therefore, it is important to develop strategies for combining sEVs with biomaterials to increase their retention in the damaged heart tissue 38. Alginate biomaterial offers an excellent promise for myocardial tissue engineering because of its practicality and pleiotropic effects. It has been shown to be biocompatible and non-immunogenic and possesses a certain degree of mechanical hardness, which is similar to natural ECM, and is, therefore, ideal for supporting infarcted heart tissue. The safety and efficacy of alginate biomaterial in preventing ventricular remodeling have been tested in clinical trials, which confirmed good tolerance with a low incidence of adverse events and preservation of left ventricular cardiac function 21, 25, 39, 40. Therefore, alginate hydrogel is a good biomaterial to combine with sEVs treatment. In our study, the combination of sEVs and the hydrogel significantly increased sEVs retention in the heart and improved the reparative potency.

The goal of the post-MI treatment is to save moribund cardiomyocytes as well as reduce cell damage and adverse ventricular remodeling. The novel approach of incorporating sEVs in alginate hydrogel offers several advantages. First, the important advantage of the sEVs-Gel is, it allows the continuous release of sEVs from the hydrogel for a few days after MI compared to the sEVs only injection, mediating a sustained anti-apoptotic effect for improving the survival of cardiac cells conducive to the recovery of cardiac function. The reason of the sustained anti-apoptosis effect is probably associated with the sEVs-Gel serving as a reservoir of anti-apoptotic miRNAs for cardioprotection, such as mir-19a and mir-21-5p 41, 42. Second, inflammatory cells, especially macrophages, are responsible for clearance of dead cells, regulation of mesenchymal cells, and cardiac remodeling. All of these processes directly affect the prognosis of myocardial infarction and heart function recovery 43, 44. Selective inhibition of M2-like protective macrophages exacerbated cardiac dysfunction and rupture after myocardial infarction confirming the importance of M2-like healing macrophages in cardiac repair 45. It has been reported that sEVs can promote macrophage polarization to the M2 type. Couto et al have shown that the exosomal transfer of miR-181b into macrophages underlies their cardioprotective effect 27. Ren et al reported that miR-21-5p delivery by MSC-EV promoted macrophage M2 polarization 46. Similarly, our data suggested that sustained release of sEVs reduced the total number of CD68+ macrophages and shifted the macrophage polarization towards the M2 phenotype, hence improved the healing response after MI. And third, angiogenesis is known to be very important for heart function recovery 47. A previous study confirmed that MSC-derived sEVs promoted angiogenesis after MI by carrying bioactive factors such as miRNA-132 48. In our study, we showed that the sustained release of sEVs by alginate hydrogel prolonged the effective time thereby enhancing the pro-angiogenesis effect compared with the short-lived effect of sEVs injection, which contributed to the better heart function in the sEVs-Gel group.

In the context of future clinical translation, safety and efficacy of an injectable hydrogel for treating MI by percutaneous trans-endocardial injection have been shown to reduce the damage of surgical procedure 49. In this study, we have developed a novel technique by incorporating sEVs within the hydrogel and demonstrated that the sustained release of MSC-derived sEVs promoted injury repair and cardiac function. This may provide a novel approach for heart repair after MI.

## Conclusion

In this study, MSC-derived sEVs were incorporated in alginate hydrogel as a sustained delivery system. Application of this system allowed retention of sEVs in the heart compared to the sEVs only injection, maintaining the local concentration of sEVs. Hence, sEVs-Gel had better effects in promoting angiogenesis, reducing cardiac apoptosis and fibrosis, enhancing scar thickness, and eventually improving cardiac function compared to the application of sEVs only.

## Abbreviations

MSCs: mesenchymal stem cells
sEVs: small extracellular vesicles
MI: myocardial infarction
ECM: extracellular matrix
TEM: transmission electron microscopy
PBS: phosphate buffer saline
SEM: scanning electron microscopy
DiR: 1,1'-Dioctadecyl-3,3,3',3'-Tetramethylindotricarbocyanine Iodide
DAPI: 4',6-diamidino-2-phenylindole
α-SMA: alpha-smooth muscle actin
HUVECs: human umbilical vein endothelial cells
SDS: sodium dodecyl dulfate
HGF: hepatocyte growth factor
VEGF: vascular endothelial growth factor
PDGF-BB: platelet derived growth factor BB
LVEF: left ventricular ejection fraction
LVFS: left ventricular fraction shortening
LVIDD: left ventricular end diastolic diameter
LVIDS: left ventricular end systolic diameter
DMEM: dulbecco's modified eagle's medium
FBS: fetal bovine serum
EDTA: ethylene diamine tetraacetic acid
OCT: optimal cutting temperature compound
