# Supplementary material for: Incorporation of small extracellular vesicles in sodium alginate hydrogel as a novel therapeutic strategy for myocardial infarction
Source: Theranostics. 2019 Oct 11;9(24):7403–16. doi: 10.7150/thno.32637 (PMC6831299; doi:10.7150/thno.32637)
Supplement: Supplementary file 1 — Supplementary figures. [file thnov09p7403s1.pdf]

Supporting Information

Incorporation of small extracellular vesicles in sodium alginate hydrogel as a novel therapeutic strategy for myocardial infarction

Kaiqi Lv[1,2]*, Qingju Li[1,2]*, Ling Zhang[1,2], Yingchao Wang[1,2], Zhiwei Zhong[1,2], Jing Zhao[1,2], Xiaoxiao Lin[3], Jingyi Wang[1,2], Keyang Zhu[1,2], Changchen Xiao[1,2], Changle Ke[1,2], Shuhan Zhong[1,2], Xianpeng Wu[1,2], Jinghai Chen[1,2,4], Hong Yu[1,2], Wei Zhu[1,2], Xiang Li[5], Ben Wang[4], Ruikang Tang[6], Jian'an Wang[1,2], Jinyu Huang[7#], Xinyang Hu[1,2#].

1 Department of Cardiology of The Second Affiliated Hospital, Zhejiang University School of Medicine, Hangzhou, PR China;

2 Cardiovascular Key Laboratory of Zhejiang Province, Hangzhou, PR China;

3 Nanjing Medical University, Nanjing, PR China;

4 Institute of Translational Medicine, Zhejiang University, Hangzhou, PR China;

5 State Key Laboratory of Silicon Materials, School of Materials Science and Engineering, Zhejiang University, Hangzhou, Zhejiang 310027, PR China;

6 Center for Biomaterials and Biopathways, Department of ChemistryState Key Laboratory of Silicon Materials, School of Materials Science and Engineering, Zhejiang University, Hangzhou, Zhejiang 310027, PR China;

7 Department of Cardiology, Affiliated Hangzhou First People's Hospital, Zhejiang University School, Hangzhou, PR China.

*These authors contributed equally to this article.

# Co-corresponding authors: hxy0507@zju.edu.cn (Xinyang Hu) ; hjyuo@163.com (Jinyu Huang)

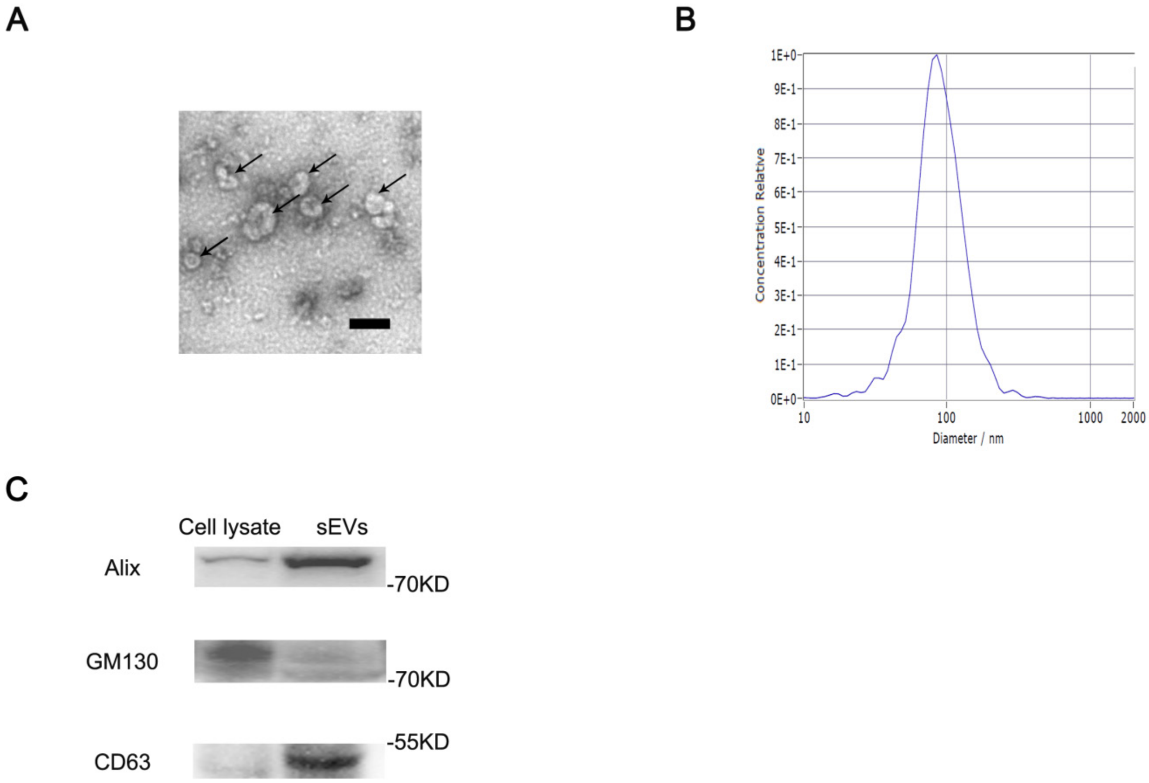

Figure S1. Identification of sEVs. (A) Transmission electron micrographs of sEVs displaying their morphology and size (bar = 100 nm). (B) ZetaView (Particle Metrix, Germany) was used to measure the particle size distribution of sEVs. (C) Western blotting was performed to characterize sEVs positive makers CD63, Alix and negative maker GM130.

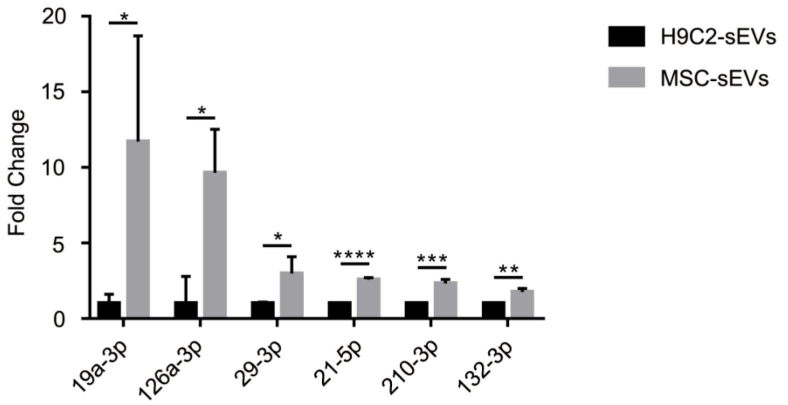

Figure S2. Expression of miRNAs in sEVs from H9C2 cells and MSCs. Comparison of mirRNAs 19a-3p, 126a-3p, 29-3p, 21-5p, 210-3p, 132-3p in sEVs from H9C2 cells and MSCs. *P < 0.05; **P < 0.01; ***P < 0.001; ****P < 0.0001.

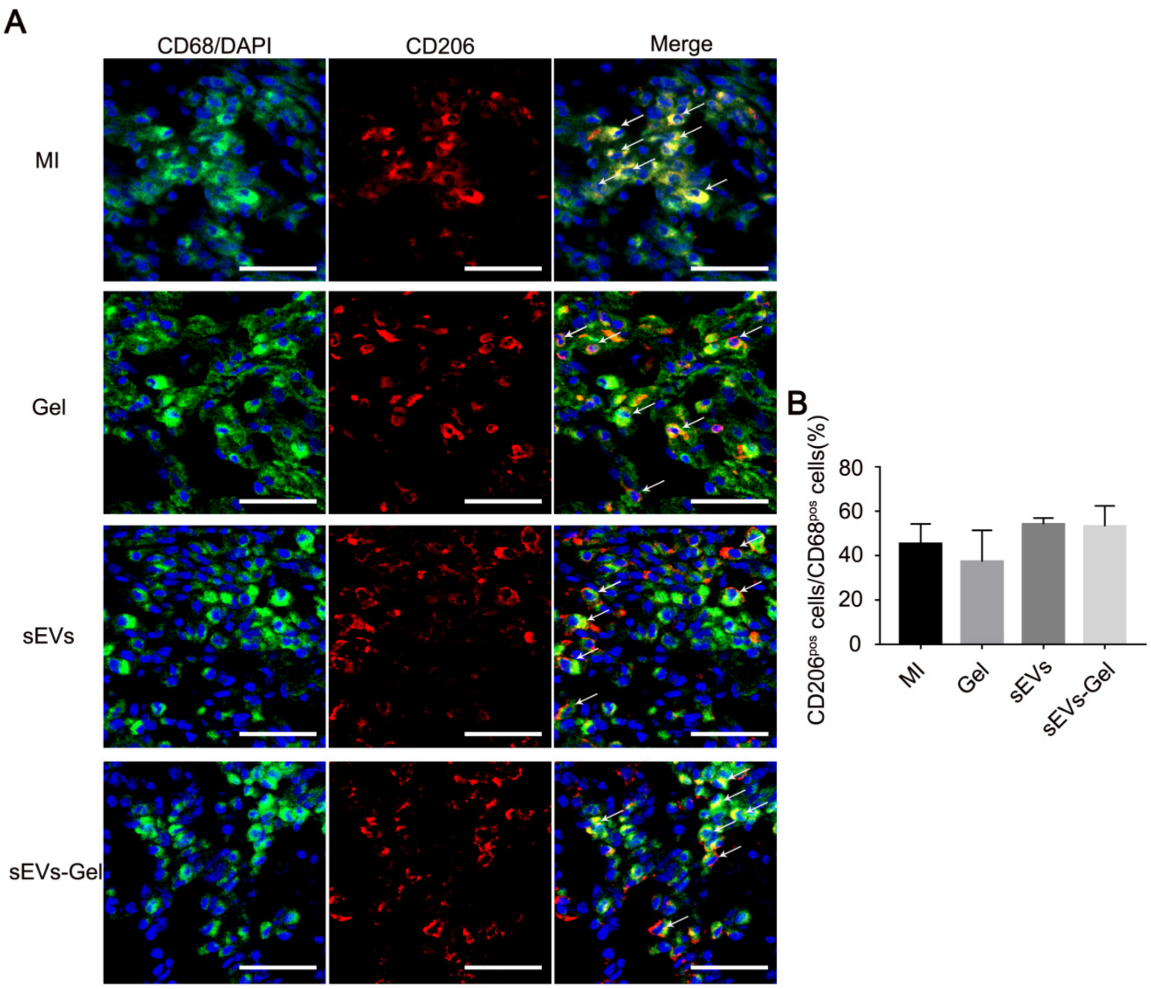

Figure S3. Evaluation of the ratio of CD206 to CD68 in the infarct area at day 7. (A) Immunofluorescence staining of CD68- and CD206-positive macrophages at day 7. Bar = 50 µm. (B) Quantitative analysis of the ratio of CD206 to CD68. n=3 for each group.

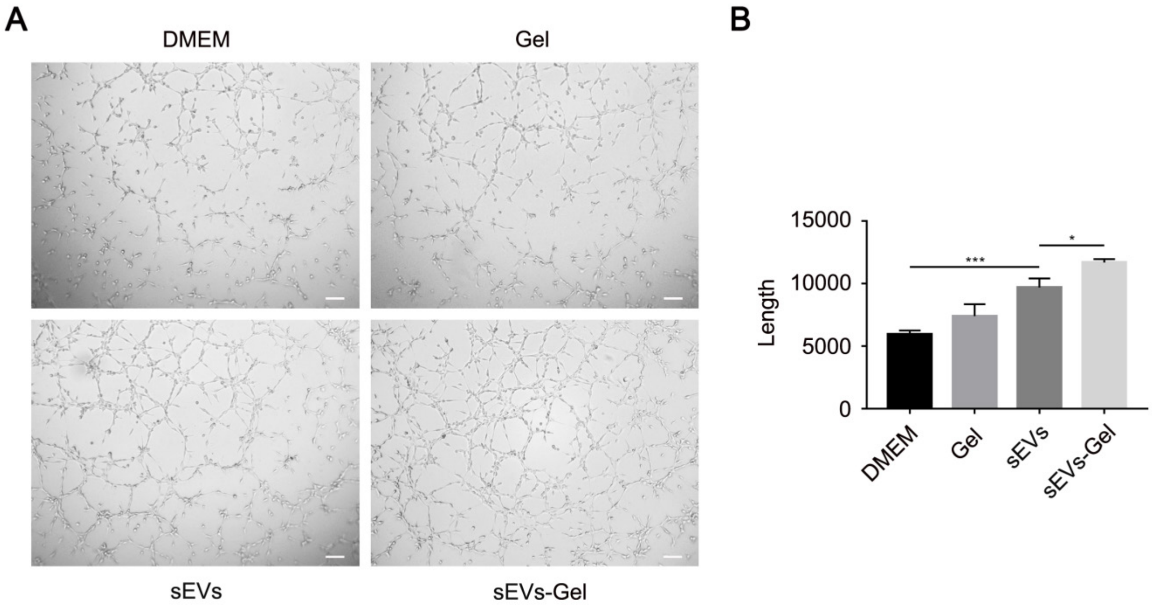

Figure S4. sEVs-Gel promotes the tube formation of HUVECs. (A) Representative tube formation ability of HUVECs with different pretreatments. Bar=100 μm. (B) Quantitative analysis of tube formation length. n=3 per group. $^{*}P < 0.05$; $^{***}P < 0.001$.

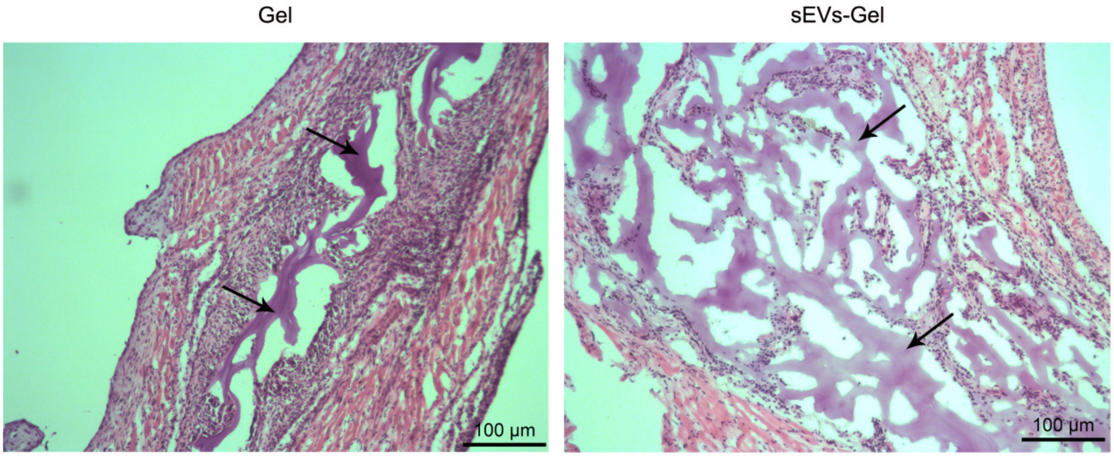

Figure S5. The residual hydrogel in the infarct area 4 weeks after MI. Representative photomicrographs of HE staining showing residual hydrogel in the sEVs and sEVs-Gel groups. Arrows point to hydrogel. Bar=100 μm.
